# Erythema Ab Igne due to Heating Pad Use: A Case Report and Review of Clinical Presentation, Prevention, and Complications

**DOI:** 10.1155/2016/1862480

**Published:** 2016-01-03

**Authors:** Marissa Milchak, Joanne Smucker, Catherine G. Chung, Elizabeth V. Seiverling

**Affiliations:** ^1^Penn State Hershey College of Medicine, Hershey, PA 17033, USA; ^2^Department of Dermatology, Penn State Hershey Medical Center, Hershey, PA 17033, USA; ^3^Department of Dermatology and Pathology, Penn State Hershey Medical Center, Hershey, PA 17033, USA

## Abstract

Erythema ab igne is an asymptomatic cutaneous condition caused by exposure to heat. Cases of erythema ab igne may prove to be diagnostically challenging due to lack of familiarity with the condition. While this dermatosis carries a favorable prognosis, nonmelanoma skin cancers have been reported to arise within lesions of erythema ab igne. Erythema ab igne is preventable, and, thus, clinicians should provide education regarding safe use of heating devices to patients using these products in both outpatient and inpatient settings.

## 1. Introduction

Erythema ab igne presents as reticular, erythematous to violaceous patches on sites of exposure to heat. Although it can present on any body surface, most cases of erythema ab igne are on the back or thighs, correlating with areas where heating pads are commonly used. Erythema ab igne can resemble numerous skin conditions including livedo reticularis, livedo racemosa, cutis marmorata, and cutis marmorata telangiectasia [1]. Although rare, nonmelanoma skin cancers may arise within sites of erythema ab igne [2, 3]. Our report aims to discuss erythema ab igne related to the use of heating pads and to provide recommendations for safe heating pad use.

## 2. Case Report

**Figure 1:**
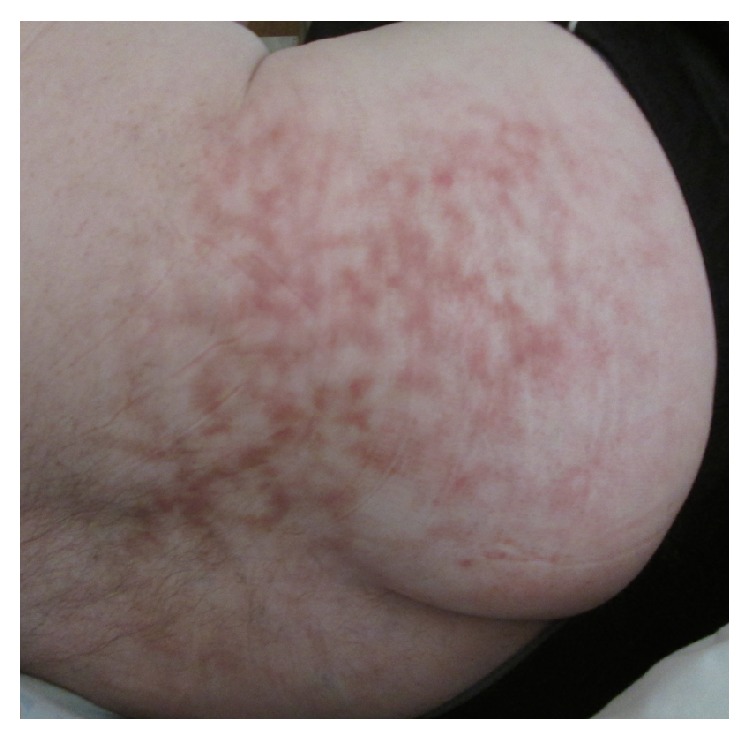
Cutaneous lesions of erythema ab igne in a hospitalized patient with a history of long-term heating pad use.

**Figure 2:**
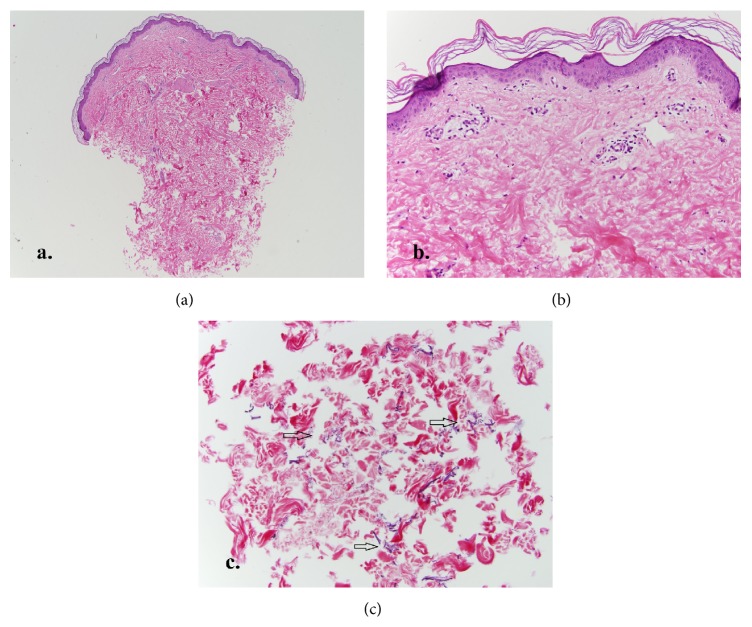
(a) Scanning magnification of a punch biopsy of lesional skin reveals a slightly attenuated epidermis, but otherwise unremarkable skin (H&E, 40x). (b) High power view reveals a nonspecific sparse perivascular lymphocytic infiltrate (H&E, 200x). (c) Elastic fiber stain demonstrates fragmented, elastic fibers in the midreticular dermis (arrows); Verhoeff-Van Gieson stain, 200x.

A 38-year-old male was admitted to the hospital for an exacerbation of previously diagnosed acute intermittent porphyria manifesting as severe abdominal, back, and lower extremity pain. He was treated with IV fluids, hemin, and oxycodone. Dermatology was consulted to assess a reticulated, dusky area on the lower back and buttocks (Figure 1). The area was not pruritic. He first noticed the skin changes 3-4 weeks prior to hospital admission. The patient had been using a heating pad on his lower back, as advised by his primary care physician, for several months in order to relieve back pain. The diagnosis of erythema ab igne was made given the patient's history of persistent skin exposure to localized heat. A punch biopsy of skin was performed (Figure 2), and histopathologic findings were supportive of erythema ab igne. The patient was counseled on safe use of heating pads and was discharged after an uncomplicated hospital course.

## 3. Discussion

Erythema ab igne presents initially as transient, blanchable erythema followed by progression to a reticular pattern of hyperpigmentation with epidermal atrophy and telangiectasias after continued heat exposure [4]. Although erythema ab igne is historically associated with exposure to open fires and wood-burning stoves, the condition is now related to more technologically advanced sources of heat such as heating pads, space heaters, heated car seats, and laptop computers [4]. Because it is caused by prolonged exposure to heat sources at temperatures insufficient to cause burns (between 37 and 113°F), erythema ab igne is typically asymptomatic [5]. Given the asymptomatic nature and frequent difficulty in visualizing the site (especially when on the back), patients with erythema ab igne may be unaware that they have developed this hyperpigmented dermatosis.

Skin biopsies may demonstrate a range of findings, from a sparse perivascular infiltrate in early lesions to epidermal atrophy, telangiectasias, keratinocyte atypia, and hemosiderin deposition. Late, well-established lesions can demonstrate increased elastin fibers, which may require special stains to identify [3]. Because histopathologic findings are generally nonspecific, skin biopsy is more helpful to exclude other differential diagnoses (such as cutaneous vasculitis) rather than to confirm a diagnosis of erythema ab igne.

While erythema ab igne carries a favorable prognosis, squamous cell carcinoma and Merkel cell carcinoma have been reported to arise within lesions of erythema ab igne [2, 3]. If lesions of erythema ab igne continue to evolve or ulcerate, biopsy should be performed to rule out malignancy. Prevention can occur both primarily with patient education and secondarily with early detection and removal of a causative heat source. Physicians recommending the use of heating pads should be aware of associated adverse cutaneous events and should counsel patients on safe practice while using these products. Proper use of heating pads should also be part of nursing and physician orders when this mode of pain relief is utilized in the inpatient setting. Safe practice guidelines recommend that heating pads be used in 15–20-minute intervals and that patients never exceed 30 minutes of use in one therapeutic session [6]. Recommendations also include that the pad be placed on top of, and not underneath, a body part, as trapped heat causes increased temperature with increased risk of development of erythema ab igne [7]. Additional information regarding specific brands of heating pads can be found on package inserts.
